# Whole-Genome Sequence of *Corynebacterium pseudotuberculosis* Strain 226, Isolated from the Abscess of a Goat in California

**DOI:** 10.1128/genomeA.00038-16

**Published:** 2016-02-25

**Authors:** Larissa M. Dias, Jorianne T. C. Alves, Adonney A. O. Veras, Rafael A. Baraúna, Pablo H. C. G. Sá, Sharon Spier, Judy M. Edman, Luis C. Guimarães, Flávia S. Rocha, Rommel T. J. Ramos, Vasco Azevedo, Artur Silva, Adriana R. Carneiro

**Affiliations:** a Federal University of Pará, Center of Genomics and System Biology, Laboratory of Genomic and Bioinformatics, Belém, Pará, Brazil; b Department of Medicine and Epidemiology, School of Veterinary Medicine, University of California, Davis, California, USA; c Institute of Biological Sciences, Federal University of Minas Gerais, Belo Horizonte, Minas Gerais, Brazil

## Abstract

*Corynebacterium pseudotuberculosis* is the etiological agent of a caseous lymphadenitis disease. Herein, we present the first complete genome sequencing of *C. pseudotuberculosis* strain 226, isolated from an abscess of the sub-iliac lymph node of a goat from California (USA). The genome contains 2,138 coding sequences (CDSs), 12 rRNAs, 49 tRNAs, and 72 pseudogenes.

## GENOME ANNOUNCEMENT

*Corynebacterium pseudotuberculosis* is a pathogenic microorganism of medical and veterinary interest and has worldwide distribution (1). The species is classified by their biochemical properties: *ovis* (negative nitrate reductase) and *equi* (positive nitrate reductase) (2). The biovar *ovis* frequently infects sheep, goats, and pigs, causing caseous lymphadenitis (CLA) (3, 4).

In goats, infection can manifest in two clinical forms: cutaneous or superficial and visceral. The first form is characterized by the formation of abscesses in the superficial lymph nodes or subcutaneous tissues (3). The other form, visceral, affects organs including the kidneys, mammary glands, uterus, liver, heart, and brain (3).

Currently, there are thirty-one complete sequences and two draft genomes of *C. pseudotuberculosis* available in the GenBank database (http://www.ncbi.nlm.nih.gov). Although, several genomes have been elucidated, virulence factors are not fully characterized.

*C. pseudotuberculosis* strain 226, biovar *ovis*, is the first complete genome isolated and sequenced from a goat in California. It was isolated from the sub-iliac lymph node of a 4-year-old LeMancha breed goat from a geographic endemic region for CLA.

The genome of *C. pseudotuberculosis* strain 226 was sequenced with the Ion Torrent PGM platform using Chip 318 and the fragment library. Sequencing processes resulted in 370,840,598 reads with an estimated genomic coverage of 158× based on the reference genome of *C. pseudotuberculosis* strain 42/02-A (accession number CP003062). The quality of raw data was analyzed with the FastQC software (http://www.bioinformatics.babraham.ac.uk), and the assembly was performed with MIRA 4.0.2 software (5), which yielded 27 contigs. Subsequently, the contigs were subjected to analysis with the SeqMan Pro tool of the Lasergene 11 Core Suite (http://www.dnastar.com) to generate a preliminary scaffold and gaps closure proceeded through CLC Genome Workbench (http://www.clcbio.com).

The genome was automatically annotated using Rapid Annotations using Subsystems Technology (RAST) (6). tRNAs and rRNAs were predicted using tRNAScan-SE (7) and RNAmmer (8) software. Additionally, the protein domain and motif were identified with the Pfam (9) and InterPro (10) databases. The signal peptides cleavages sites, transmembrane helices and CRISPR repeats were predicted by the SignalP 4.1 server (11), TMHMM Server v2.0 (http://www.cbs.dtu.dk/services/TMHMM/), and CRISPRfinder (12). Artemis program (13) was used to manual curation of the annotation. Pathogenicity islands (PAIs) was performed by the PIPS (14) software using the genome of nonpathogenic *C. glutamicum* strain ATCC13032 (accession number BA000036). The parameters utilized were a G+C content of 1.5 standard deviations, 95% codon usage deviation, and e-value of 10^−5^ to factors virulence.

The genome of *C. pseudotuberculosis* strain 226 contains 2,337,820 bp and with a G+C content of 52.18%. A total of 2,271 genes were predicted with 2,138 coding sequences (CDSs), 49 tRNAs, 4 cluster rRNAs, and 72 pseudogenes. We identified 151 signal peptides, 595 proteins as transmembrane helices, and 1 CRISPR-associated protein (*Cas5* gene). This genome has eight PAIs with 112 genes, of which, 37 are hypothetical proteins and can be related to the virulence of the bacterium.

### Nucleotide sequence accession number.

The genome project has been deposited in GenBank under the accession number CP010889.
